# A new microchannel capillary flow assay (MCFA) platform with lyophilized chemiluminescence reagents for a smartphone-based POCT detecting malaria

**DOI:** 10.1038/s41378-019-0108-8

**Published:** 2020-01-27

**Authors:** Sthitodhi Ghosh, Kashish Aggarwal, Vinitha T. U., Thinh Nguyen, Jungyoup Han, Chong H. Ahn

**Affiliations:** 1 Department of Electrical Engineering and Computer Science, Microsystems and BioMEMS Laboratory, University of Cincinnati, Cincinnati, OH 45221 USA; 2 Mico BioMed USA Inc., 10999 Reed Hartman Highway, STE 309C, Cincinnati, OH 45242 USA

**Keywords:** Biosensors, Microfluidics

## Abstract

There has been a considerable development in microfluidic based immunodiagnostics over the past few years which has greatly favored the growth of novel point-of-care-testing (POCT). However, the realization of an inexpensive, low-power POCT needs cheap and disposable microfluidic devices that can perform autonomously with minimum user intervention. This work, for the first time, reports the development of a new microchannel capillary flow assay (MCFA) platform that can perform chemiluminescence based ELISA with lyophilized chemiluminescent reagents. This new MCFA platform exploits the ultra-high sensitivity of chemiluminescent detection while eliminating the shortcomings associated with liquid reagent handling, control of assay sequence and user intervention. The functionally designed microchannels along with adequate hydrophilicity produce a sequential flow of assay reagents and autonomously performs the ultra-high sensitive chemiluminescence based ELISA for the detection of malaria biomarker such as PfHRP2. The MCFA platform with no external flow control and simple chemiluminescence detection can easily communicate with smartphone via USB-OTG port using a custom-designed optical detector. The use of the smartphone for display, data transfer, storage and analysis, as well as the source of power allows the development of a smartphone based POCT analyzer for disease diagnostics. This paper reports a limit of detection (LOD) of 8 ng/mL by the smartphone analyzer which is sensitive enough to detect active malarial infection. The MCFA platform developed with the smartphone analyzer can be easily customized for different biomarkers, so a hand-held POCT for various infectious diseases can be envisaged with full networking capability at low cost.

## Microfluidic Platforms: Smartphone-based point-of-care testing for malaria

A smartphone-based point-of-care-testing (POCT) analyzer using a microfluidic platform has been developed for detecting infectious diseases, such as malaria. A major challenge to health care has been a lack of simplified, inexpensive POCT analyzers that can quantitatively detect low concentrations of target biomarkers in disease diagnosis. A team headed by Chong H. Ahn at the University of Cincinnati, United States has developed a handheld POCT system for detecting infectious diseases that uses a smartphone for display, data transfer, storage, and analysis. Their system employs a novel microfluidic-based platform, which utilizes the ultra-high sensitivity of chemiluminescent detection: it was sufficiently sensitive to detect active malarial infection. The authors believe their microfluidic platform and smartphone analyzer can be easily customized for different biomarkers, permitting low-cost handheld POCT systems to be applied for various infectious diseases.

## Graphical abstract

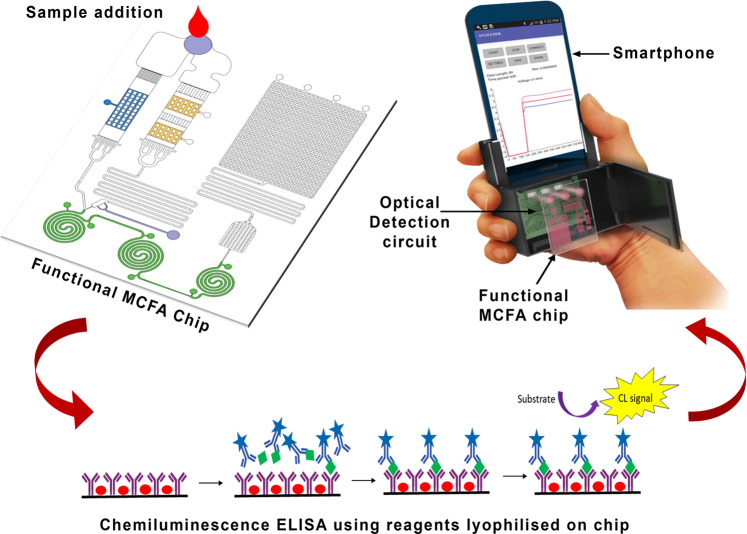

## Introduction

One of the biggest challenges in the fast growing healthcare industry is a dearth of simplified point-of-care testing (POCT) systems that can quantitatively detect low concentrations of a target biomarker in biological fluids for disease diagnosis in resource limited settings^1,2^. A huge percentage of deaths related to major infectious diseases occur in resource-poor countries that have limited access to clinical laboratory facilities and trained personnel^3^. Developing reliable diagnostic tests that can be used at the point-of-care can result in earlier disease diagnosis, improved patient treatment, and more efficient outbreak prevention. The emergence of miniaturization and microfluidics has led to the development of cheap and novel lab-on-a-chip (LOC) platforms for rapid and sensitive immunodiagnostics. Microfluidic assays have been reported to have several advantages over conventional immunodiagnostics methods. Smaller device size, reduced sample volume, portability, faster detection and higher sensitivity are some of the many^4–7^ benefits which make LOC-based assays a well-established component of POCT systems.

An ideal POCT also requires the development of a simplified user-friendly analyzer that can ably perform operations like data acquisition, data analysis, data transmission, display and storage which often results in bulky and expensive instruments. In recent years, the idea of employing smartphone as a POCT analyzer has become popular due to the ubiquitous presence of smartphone in the present world^8^. As of 2018, there were 2.51 billion smartphone subscribers worldwide with an unprecedented growth in the developing countries. Smartphone incorporates all the basic features of an electronic system such as a touch-controlled screen, high-speed processor, communication ports (USB, Audio jack), wireless connectivity (Bluetooth and Wi-Fi), storage media (internal memory, SD card) and rechargeable battery which makes it an ideal choice for a POCT analyzer, especially in developing countries that lack adequate resources and laboratory facilities. Utilizing smartphone’s camera as an optical sensor and performing digital image processing on the phone has been quite popular recently^9–11^. However, smartphone camera on its own is still not efficient enough for ultra-high sensitive optical detection, as required in immunodiagnostics. It requires use of more sensitive optical detectors such as photomultiplier tubes or photodiodes. Thus, it is better to have an external high-sensitive optical detector which is attachable to the smartphone through its communication ports (e.g., audio jack, USB port, bluetooth) instead of using the phone’s inbuilt sensors. A number of systems have been reported that uses a smartphone’s audio jack to derive the power from the smartphone and performs data communication through audio jack channels^12,13^. A disadvantage of extracting power from the audio port is in terms of maximum voltage that is available from the audio jack which is very low^14^ and varies depending on the smartphones. It needs to be rectified and boosted by a harvester circuit, adding to the cost and complexity of the system^15,16^. Thus, in this work, the USB On-The-Go (OTG) port of the smartphone is chosen as the communication port for an attachable/detachable optical detector. This port works for communications as well as provides power to the optical detector. The USB port on a smartphone can provide 4.4–5.25 V for a load of less than or equal to 100 mA, and 4.75–5.25 V for a load greater than 100 mA. Along with providing power, USB On-The-Go (OTG) also provides the capability of digital data communication between the smartphone and an external device and have been successfully utilized to develop a mobile phone-based platform for quantitative biomolecular detection^17,18^.

Considering the low power that can be extracted from the smartphone via the USB-OTG protocol, a new functional microfluidic lab chip with passive flow control needs to be developed. Accordingly, the strip-based lateral flow immunoassay (LFIA) chips relying on capillary flow seems like an ideal solution. Although the LFIA strips have been one of the most popular immunoassay kits, they suffer from inconsistencies due to poor sensitivity, often resulting in poor diagnosis^19,20^. Most of the LFIA strips provide qualitative (true/false) or visual readouts causing difficulty and vagueness in data interpretation^21^. Microchannel-based immunoassay on polymer substrate implementing enzyme-based signal amplification can largely improve the limit of detection (LOD) of the microfluidic chips and can overcome the disadvantages associated with strip-based LFIA. However, the reported POCT systems performing sandwich enzyme linked immunosorbent assay (ELISA)^22–24^ in microfluidic formats involve multiple liquid handing steps which demands the participation of trained users. The inconvenient user intervention can be minimized by drying the assay reagents on chip prior to sample addition which can lead to an ideal sample-to-answer POCT platform. The use of dry reagents reduces user steps, removes the need for a cold chain, provides a longer shelf life for the test, and facilitates device automation^25^. Lyophilization or freeze-drying is an established method of drying biological reagents^26^ and POCT devices with on-chip lyophilized reagents have become popular. The Biosite Triage^27^ is capable of multiplexed quantitative detection of 3 cardiac markers within 15 min using 150 μL of sample. PDMS based chip for one step immunoassay has been reported by Delamarche et al.^28^. Automated microfluidic on-card assays using dried reagents has also been reported^29,30^.

Majority of the reported autonomous microfluidic assays using dry reagents have employed fluorescence-based detection method^27–29^ due to the simplicity of assay protocol. However, fluorescence detection requires an optical excitation source which in turn requires more power making the optical detection system costly and complicated. In contrast, chemiluminescence based ELISA relying on the reaction between enzyme (e.g., Horseradish Peroxidase (HRP)) and chemiluminescent substrate has been shown to produce an ultra- high detection sensitivity^22,31^. Measurement of chemiluminescence is also relatively simple, requiring only a photomultiplier or photodiode and the associated electronics to convert and record signals. Despite having a higher sensitivity as compared to colorimetric or fluorescent based detection^32,33^ and a simplified detection protocol, dry reagent based chemiluminescence assay has rarely been reported. The successful drying of chemiluminescent substrate reagents, as validated in our previous work^34^ paved the way towards performing dry reagent based chemiluminescence assay on microfluidic chip. Referring to the different functional components of reported capillary microfluidic systems^28,35–37^, a new microchannel capillary flow assay (MCFA) lab chip was proposed and developed in this work, which can reliably perform chemiluminescence based sandwich ELISA.

**Fig. 1:**
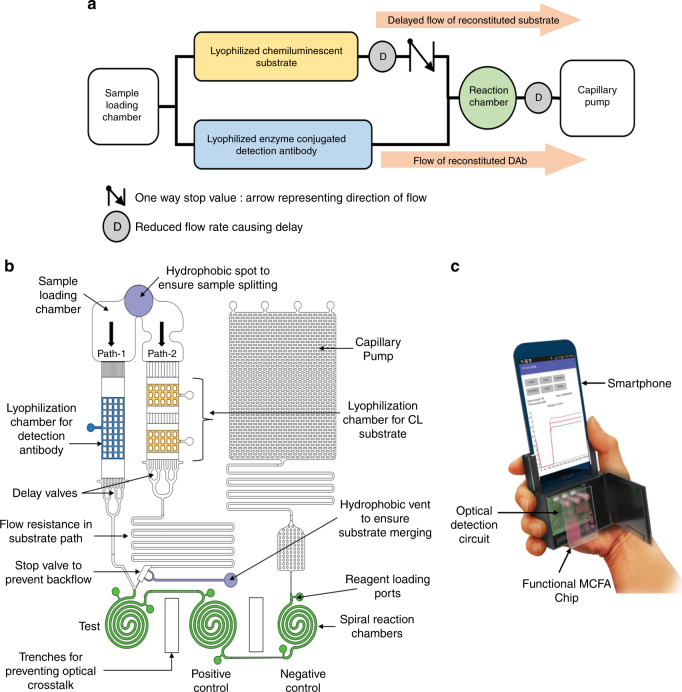
Overview of the smartphone based POCT analyzer. **a** Functional concept of the MCFA lab chip with on-chip reservoirs in operation sequence to implement chemiluminescence based sandwich ELISA, **b** Schematic of the designed MCFA chip with detailed labeling of the microfluidic components and **c** Schematic of the developed smartphone-based POCT analyzer

The functional concept of the new MCFA lab chip is shown in Fig. 1a. On addition of the sample (serum) containing the target biomarker, the functionally designed microchannels coupled with adequately hydrophilic surfaces pull the sample towards the dried reagents and reconstitutes them. The designed microfluidic chip with two parallel paths allows the antigen-detection antibody complex to bind with the immobilized capture antibody in the reaction chambers prior to the arrival of the reconstituted substrate and maintains the required sequence of sandwich ELISA. The reconstituted substrate reacts with the immobilized complex producing the desired chemiluminescent light which can be correlated to the target biomarker concentration. The immunoassay performed on the chip is entirely controlled by channel geometry and surface properties without any external pumping and the device will be newly referred as the microchannel capillary flow assay (MCFA) lab chip for the rest of the paper. The detailed design of the MCFA lab chip is shown in Fig. 1b.

This paper also reports the design and development of a smartphone based POCT analyzer for performing chemiluminescence based sandwich ELISA on preloaded MCFA lab chip. A schematic of the overall smartphone-based analyzer systems is shown in Fig. 1c. The whole system consists of 3 primary components: (a) the MCFA lab chip, made of thermoplastic Cyclic Olefin Copolymer (COC) provides a cheap and disposable platform for performing high sensitive chemiluminescence assay, (b) the high-sensitive optical detector (e.g., attachable and detachable to the smartphone) that detects the chemiluminescent signal from the chip and communicates with the smartphone in real-time via the USB on-the-go (OTG) protocol and (c) the smartphone analyzer which is used for display, data transfer, data storage and analysis, and also works as the power source for the optical detector. The USB-OTG feature enables the smartphone to act as a host and power the peripheral device connected to it. The developed whole system in this work is portable, user friendly, can perform fast and sensitive measurements and can be viewed as an ideal POCT analyser with minimum intervention of end-users. The diagnostic ability of the system developed in this work was validated by implementing a sandwich assay on the MCFA lab chip for the detection of malarial biomarker Plasmodium falciparum histidine rich protein 2 (PfHRP2) as a demonstration vehicle. The PfHRP2 is regarded as a vital biomarker unique to P. falciparum virus and causes the most severe form of malaria^38^. As evidence shows that PfHRP2-based assays are more sensitive towards detection of P. falciparum than aldolase- and LDH-based diagnostic tests^39^, it was used as the principle biomarker in this work. PfHRP2 was detected in artificial serum at concentrations as low as 8 ng/mL which was enough for the validation of diagnostics. Each test, which involved a sample loading step and a vent opening step, can be completed within 20 min after sample loading. The developed POCT based on smartphone thus greatly reduces the time and complexity associated with disease diagnostics and for the first time a new concept of microchannel capillary flow assay (MCFA) platform using lyophilized chemiluminescence substrate has been fully developed and reported in this work.

## Results and discussion

### Design of the MCFA lab chip

The reported MCFA lab chips were designed for single-step sample loading and capillary liquid transport to initiate a chemiluminescence based sandwich ELISA in the reaction chambers. The lab chip is an ensemble of connected microstructures through which the sample flows seamlessly by means of capillary flow. The flow rate *Q* of a liquid at any point in the MCFA lab chip can be determined by the wettability of the channel surfaces, the viscosity of the liquid, the total flow resistance and the capillary pressure at the point and can be expressed as:

1 $$Q = \frac{{{\it{\Delta }}P}}{{\eta R_F}}$$

where *η* is the viscosity of the liquid, *ΔP* is the difference in capillary pressure and *R*_*F*_ is the total resistance to flow of the flow path. The resulting capillary pressure *P*_*cap*_ of a liquid–air meniscus in such a rectangular microchannel is

2 $$P_{{\it{cap}}} = - {\it{\gamma }}\left\{ {\frac{{{\it{cos}}\theta _{{\it{top}}} + {\it{cos}}\theta _{{\it{bottom}}}}}{h} + \frac{{{\it{cos}}\theta _{{\it{left}}} + {\it{cos}}\theta _{{\it{right}}}}}{w}} \right\}$$

where *γ* is the surface tension of the flowing liquid, *θ*_*top*_, *θ*_*bottom*_,*θ*_*left*_, *θ*_*right*_ are the contact angles of the liquid on the four channel walls, and *h* and *w* are the depth and width of the microchannel, respectively. The negative capillary pressure is similar to a suction force that pulls the fluid and controls the movement. A lower contact angle (hydrophilic surfaces) increases the capillary pressure and facilitates the flow of the fluid whereas a contact angle greater than 90° (hydrophobic surface) turns the pressure positive and prevents the forward movement. As evident from Eq. (2), the capillary pressure also depends on the channel dimensions and thus fluid movement can be controlled by careful design of the microchannels. In order to ensure a smooth flow of the sample, the microchannels were so designed that there was an increase of capillary pressure along the flow path. The sandwich ELISA protocol requires the capture antibody–antigen-detection antibody complex to form prior to the introduction of the substrate to ensure specific enzyme-substrate reaction in the reaction chambers. Accordingly, the MCFA lab chip was designed to have 2 parallel paths, path-1 which incorporates the HRP labeled detection antibody drying chamber and path-2 which has the substrate drying chamber as reported in our previous work^34^.

The overall schematic of the MCFA lab chip is shown in Fig. 1b. The MCFA lab chip includes a sample loading chamber, a detection antibody drying chamber, chemiluminescent substrate drying chamber, delay valves, a stop valve, a plurality of spiral reaction chambers and a capillary pump. The hydrophobic patch created in the sample loading chamber splits the sample into two distinct paths and facilitates the sequence of flow. A set of capillary channels are placed in between the sample loading chamber and the lyophilization chambers in both the parallel paths. The sample reservoir is designed to have a volume of around 30 µl and is in direct fluidic contact with the narrow capillary channels which prevent the diffusion of reconstituted mixture back into the sample reservoir and prevents any unwanted enzyme-substrate reaction. The surface of each of the lyophilization chambers include micropillar structures to facilitate uniform drying or lyophilization. In absence of the posts, the fluid to be dried tend to form larger menisci at the corners and produce a non-uniform layer of dried reagent^24^. The presence of the textures causes the creation of numerous small menisci and results in uniform drying. The lyophilization chambers were also designed to be confined within a descending ramp and an ascending ramp. These ramps help in confining the liquid reagents within the chamber during lyophilization process and also delay the flow of the sample to the next step ensuring better reconstitution in the drying chambers.

**Fig. 2:**
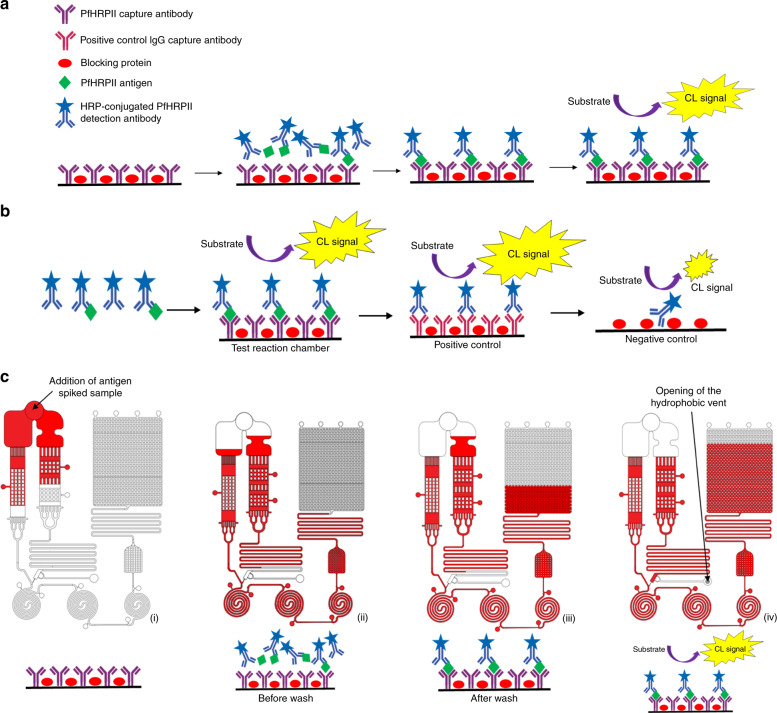
Immunoassay protocol and working principle of the MCFA chip. **a** Schematic of the immunoassay protocol for chemiluminescence based sandwich ELISA as implemented on the lab chip, **b** Schematic of the immunoassay reactions occurring in the test reaction chamber, positive control chamber and negative control chamber (from left to right) and **c** Sequential working principle of the MCFA device where (i) shows the empty test chamber immobilized with capture antibody, (ii) shows the condition when HRP-DAb-antigen complex reaches the test chamber, (iii) depicts the formation of Cab-antigen-DAb complex after adequate washing and (iv) illustrates the condition when the substrate reaches the test chamber producing chemiluminescence

Both the lyophilization chambers are followed by a series of delay valves as reported by Zimmerman et al.^40^. Such delay valves retard one of at least two filling fronts by increasing the size of the meniscus of the filling front and thereby reduce its capillary pressure. The delay valves thus help in merging a wide filling front of liquid into a single microchannel without allowing for the formation of air bubbles. It also retards the fluid flow and ensures better reconstitution in the lyophilization chambers. The meandering channel in path-2 provides a high flow resistance that delays the flow in path-2 and allows the antigen-detection antibody complex in the path-1 to flow and bind with the immobilized capture antibody in the reaction chambers. A hydrophobic vent connected to path-2, when opened, helps in the escape of the trapped air and allows the reconstituted substrate to reach the reaction chamber causing chemiluminescence. The immunoassay protocol for chemiluminescence based sandwich ELISA is shown in Fig. 2a. The micro-pillared reservoir and the meandering channel following the reaction chambers slow down the flow rate and provide longer reaction time between the antibodies and target antigen and increase the assay sensitivity. The capillary pump is designed to have a very high capillary pressure that facilitates washing of unbound reagents and waste collection. Since crosstalk and optical interference are a major drawback of chemiluminescence-based detection in optically transparent materials, optical trenches were added in between spiral reaction channels to ensure minimum crosstalk and superior assay performance. Dimensions and volume of MCFA lab chip channels and reservoirs are summarized in Table 1. Three reaction chambers were designed to act as test reaction chamber, positive control and negative control to perform quality control on the MCFA lab chip. The immunoassay reactions occurring on the three reaction chambers is explained in Fig. 2b. The chemiluminescence signal obtained from the test chamber can be directly correlated to the biomarker concentration whereas the positive control validates the functionality of the HRP-conjugated Dab. The negative control provides the blank for each operation ensuring chip validation. The microfluidic channels were designed to obtain different capillary pressure at different areas to enable a seamless flow of the sample from the loading chamber to the capillary pump while maintaining the sequence of chemiluminescence based sandwich assay as depicted in Fig. 2c. Based on the test reaction chamber volume of 2.5 μl, the lyophilization chambers, the sample loading chamber and the capillary pump were designed to have sufficient volumes to facilitate the immunoassay sequence.

**Table 1:** Dimensions and volume of the channels of MCFA lab chip

Chambers in LOC	Width (mm)	Height (mm)	2D Surface Area (mm^2^)	Volume (µl)
Sample loading chamber	NA	0.45	67.34	30
Detection antibody drying chamber (including ramp and excluding posts)	2.5	0.4	8.44	6
Substrate drying chamber (each including ramp and excluding posts)	3.5	0.4	0.3	3.5
Path-2 flow resistance	0.2	0.2	19.80	3.9
Spiral reaction chamber (test and positive control)	0.2	0.2	11.35	2.5
Spiral reaction chamber (negative control)	0.2	0.2	9.41	1.8
Post reaction chamber flow resistance	0.2	0.1	17.24	1.72
Capillary pump	NA	0.1	120.8	12

### Stop-valve design and simulation

The capillary stop valve is a key feature of the MCFA lab chip which prevents the fluid in path 1 to enter into the substrate path and allows the antigen-DAb-HRP complex to flow into the reaction chambers. The capillary pressure is dependent on the curvature of filling front at the liquid–solid–gas interface and can be stopped by causing a sudden enlargement of the front meniscus. The curvature of the meniscus in a straight channel can be represented by *α* *=* *π/2 – θ* where *θ* is the contact angle of the liquid with the channel walls. It can be shown that at an abrupt enlargement having an angle *β* between the old and new direction of the microchannel wall, the meniscus changes its curvature^41^. For higher values of *β*, the curvature becomes negative and an opposing pressure *P* is generated. The pressure barrier can be expressed as:

3 $${\it{\Delta }}P = \frac{{2\gamma _{{\mathrm{la}}}}}{w}\left\{ {\frac{{\cos \theta - \frac{\alpha }{{\sin \alpha }}\sin \beta }}{{\cos \beta + \frac{{\sin \beta }}{{\sin \alpha }}\left( {\frac{\alpha }{{\sin \alpha }} - \cos \alpha } \right)}}} \right\}$$

where *γ*_*la*_ is the surface tension of liquid–air interface and was assumed to be 73 mN/m and *w* is the width of the microchannel. Considering a contact angle of 12° which can be actually obtained, it can be shown that a pressure barrier of around 800 Pa can be generated with 50 μm of *w* and 90° of *β*. A higher value of *β* also helps to prevent creeping flow and increases the robustness of the valve.

**Fig. 3:**
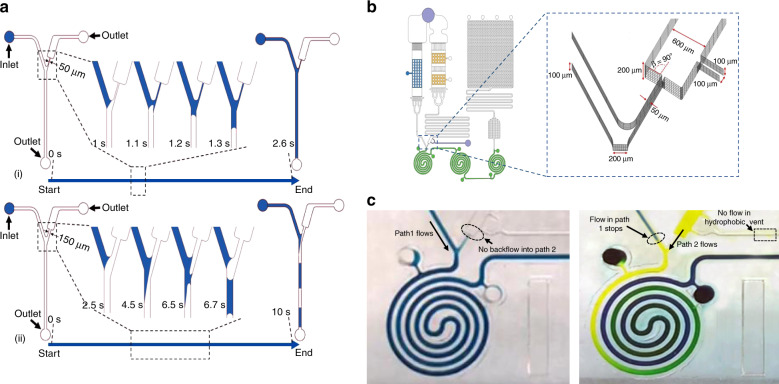
Design and simulation of the stop valve. **a** Two-phase flow level set simulation result of the stop valve area. Water is presented by blue and air is presented by white. (i) 50 μm channel connected to the stop valve and (ii) 150 μm channel connected to the stop valve (the leftmost figure shows the beginning of the flow; the rightmost figure shows the ending of the flow; 4 inset figures shows the intermediate flow and the bubble formation in case of 150 μm channel), **b** The actual design of the stop valve with dimensions and **c** Stop valve operation on the fabricated chip. Left image shows the flow of Blue dye (path-1) first into the reaction chamber with the stop valve preventing its flow into the substrate path. Right image shows the flow of yellow dye (path-2) into the reaction chamber with the opening of the vent

To confirm the proper functioning of the stop valve, a numerical fluidic simulation was performed to study the capillary flow in the merging region using COMSOL Multiphysics (Version v.5.3.a). The moving interface of the two-phase flow system was solved by using the level set method^42^. The first fluid phase was air and the second fluid phase was chosen as water. Simulations were run on multiple channel designs with the width of the microchannel connected to the stop valve varying from 50 μm (Fig. 3a(i)) to 150 μm (Fig. 3a(ii)). The contact angle of the wetted wall was set constant at 12°. The angle of enlargement *β* was fixed to be 90°. From the simulation results it is evident that the designed valve prevented the backflow into the substrate path while the flow continued in the alternate path. By increasing the length of the alternate path, it was confirmed that there was almost no leakage in the stop valve until the flow in the alternate path is impeded. However, it was also evident from the simulation, that the width of the microchannel connecting the stop valve is critical in obtaining a smooth and unimpeded flow. The 50 μm channel can instantaneously pull the fluid till the point where the channel dimensions are changed and allows for a smooth and continuous motion. However, on increasing the width, creeping flow along the channel walls become more and more prominent producing air bubbles along the flow. The bubble formation process was shown in the inset of Fig. 3a(ii) from 2.5 s to 6.7 s timestamp.

Based on the numerical analysis and simulation, the stop valve was designed, as shown in Fig. 3b. As shown from the figure, an enlargement was designed in terms of both width and height with the channel depth steeply increasing from 100 μm to 200 μm. This further increased the pressure barrier and prevented back flow. The design also facilitates the fluid to flow from the other direction. The horse-shoe shaped vent was designed to facilitate the venting of trapped air by providing 2 outlets instead of one. The enhanced hydrophobicity of the vent channel prevents the reconstituted substrate from flowing into the channel and block the venting operation. The images shown in Fig. 3c clearly shows the stop valve action and the functioning of the hydrophobic patch in controlling the fluid flow after vent opening as performed on the fabricated chip. The successful functioning of the stop valve connected to a horse-shoe shaped hydrophobic vent was critical for the implementation of the dry reagent based chemiluminescence assay.

### Assay reagent optimization

**Fig. 4:**
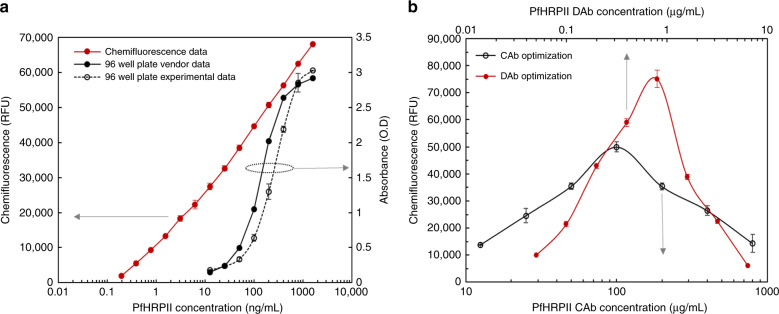
Optimization results of the assay reagents. **a** 96 well plate assay results plotted on the right axis and Chemifluorescence assay result obtained from Optimizer™ microfluidic microplate is plotted on the left axis showing lower LOD and higher dynamic range and **b** Chemifluorescence signal output variation with increase in PfHRP2 capture antibody (CAb) concentration (bottom axis) and increase in PfHRP2 detection antibody (DAb-HRP) concentration (top axis) at a fixed DAb-HRP concentration (0.1 μg/mL) and at a fixed CAb concentration (100.0 μg/mL), respectively. Each point represents the mean of three replicates

As a first step of reagent functionality validation, Malaria PfHRP2 sandwich ELISA was performed on a conventional 96-well plate following the vendor protocol. The standard curve (antigen concentration ranging from 12.5 ng/mL to 1600.0 ng/mL, with two-fold dilution in assay buffer) generated showed a typical linear trend similar to the vendor provided reference graph as shown in Fig. 4a. After confirming reagent functionality with conventional 96-well plate assay, Optimiser™ microfluidic microplates were used to determine the optimal antibody concentrations. The unique microchannel geometry of the Optimiser™ microfluidic microplate offers several important advantages, vital for development of microchannel based immunoassay^7^. Since Optimiser™ microfluidic microplates provide a similar environment as the designed microchannels on the MCFA lab chip, it acts as an excellent precursor to perform CL assay on LOC. Moreover, since the sensitivity of CL-based assays has been reported to be better than CF-based detection, the optimal antibody concentration obtained by performing chemifluorescence (CF) assay on the Optimiser^TM^ microfluidic microplates can be reliably used for CL based assay on the MCFA lab chip^43,44^. Hence, the optimal antibody concentrations obtained by performing CF assay on the Optimiser™ microfluidic microplate were used for CL based assay on LOC.

The difference in surface-to-volume ratio between a standard 96 well and the designed microchannels requires optimization of the reagent concentrations. Finding the optimal capture (CAb) and detection (DAb) antibody concentrations is critically important for translating the well-established conventional 96-well plate assay to fit the LOC. For optimizing capture antibody, DAb concentration was fixed at 0.1 µg/mL and the CAb concentration was varied from 12.5 µg/mL to 800 µg/mL. As shown in Fig. 4b, depicted by the black line, the highest CF signal corresponded to a CAb concentration of 100.0 µg/mL and gradually decreased with further increase in the CAb concentration due to the ‘hook effect’^45^. For optimizing detection antibody, CAb concentration was fixed at the optimized value, 100.0 µg/mL, and the DAb concentration was varied from 0.05 µg/mL to 6.4 µg/mL, as depicted by the red line in Fig. 4b. The highest CF signal corresponded to a DAb concentration of 0.8 µg/mL, and again gradually decreased (i.e., hook effect) with further increase in the DAb concentration. The higher CF signal obtained in DAb optimization experiment is due to the usage of optimized CAb concentration (100.0 µg/mL) in performing those assays whereas in the case of CAb optimization experiment, DAb concentration was fixed at a non-optimized concentration of 0.1 µg/mL. Similar to the above experiments, positive control capture antibody was also optimized to be 40.0 µg/mL for validating DAb functionality during on-chip assay. Microfluidic channels have key advantages of high surface-to-volume ratio and low sample volume, which increases the probability and extent of antigen binding by increasing antibody concentrations. Once antibody concentrations were optimized, Malaria PfHRP2 concentration ranging from 0.2 ng/mL to 4 ng/mL were detected using optimal CAb (100.0 µg/mL) and DAb (0.8 µg/mL) concentrations in Optimiser™ microfluidic microplate. Assay results are shown in Fig. 4a, depicted by the red line. As evident from Fig. 4a, the assay sensitivity increases by almost 2 orders of magnitude when performed in the microfluidic environment. A significant increase in the linear range of assay performance was also noted. Thus, the sandwich ELISA protocol was successfully optimized using the Optimiser™ microfluidic microplate to obtain maximum sensitivity on the developed LOC.

### Dry assay on the MCFA lab chip

The chemiluminescence method of detection relies on the enzyme-substrate reaction between the enzyme Horseradish peroxidase (HRP) and the chemiluminescent substrate. The reaction leads to the formation of luminol oxide which generates the 3-amidogen-dimethyl phthalate ion in an excited state and during the transition from the excited to the ground state emit light with a maximum wavelength of 425 nm^46^. A methodology towards successful lyophilization of chemiluminescent substrate (a mixture of enhancer and peroxide) while restoring the substrate functionality was reported in our previous work^34^. The paper also demonstrates the ability of the chemiluminescent substrate to retain their functionality when reconstituted in artificial serum and is an important step towards developing a sample-to-answer type POC system using chemiluminescence based sandwich ELISA. However, the separate drying and reconstitution of the chemiluminescent substrate components on a microfluidic chip introduces uncertainty in proper mixing of the enhancer and peroxide and affects the overall assay performance.

**Fig. 5:**
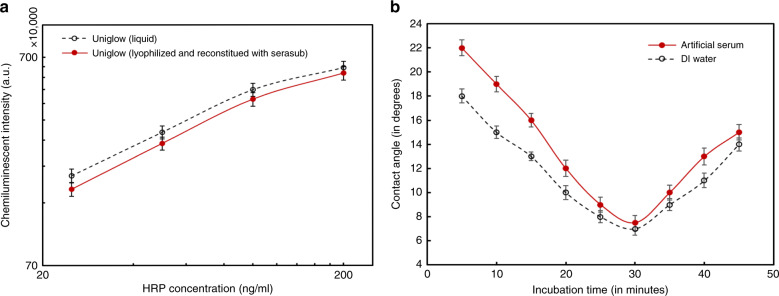
Substrate Lyophilization and BSA optimization results. **a** Variation in chemiluminescence intensity with HRP concentration for liquid chemiluminescent substrate and lyophilized and serum-reconstituted chemiluminescent substrate and **b** Contact angle analysis of DI water and artificial serum on flat COC chip, dip-coated in 1% BSA in PBS for different periods of time

**Fig. 6:**
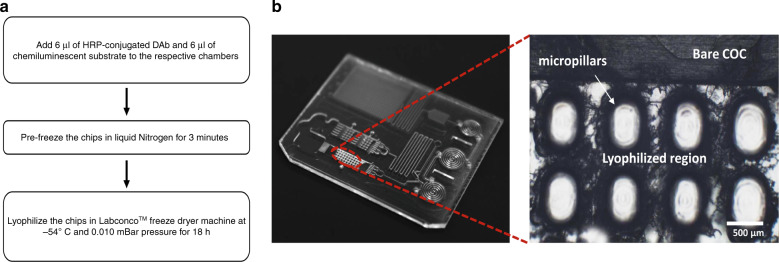
Lyophilization protocol and characterization. **a** Flowchart depicting the steps for on-chip Lyophilization and **b** Left: COC MCFA lab chip with reagents freeze-dried in the lyophilization chamber; Right: Magnified view of the DAb lyophilization chamber clearly showing the region covered by lyophilized reagents

Hence, in this work a single component ready-to-use chemiluminescent substrate (Uniglow-0100) was characterized post lyophilization and reconstitution following the same protocol as reported earlier^34^. The substrate was aliquoted to a volume of 50 μl in 1.5 mL microcentrifuge tubes, were first pre-frozen in liquid nitrogen for 5 min and then were freeze dried in Labconco^TM^ freeze-dryer system at −54 °C and 0.010 mbar pressure for 18 h. The lyophilized powder was reconstituted in artificial serum (Serasub) and used for functionality validation. Streptavidin conjugated to Horseradish Peroxidase (DY998, R&D Systems, MN, USA) was serially diluted in 1% BSA in PBS (DY995, R&D Systems, MN, USA) to obtain 4 different concentrations ranging from 25 ng/mL to 200 ng/mL. The HRP-substrate reaction was performed in the spiral reaction chambers fabricated on injection molded COC chips. The chemiluminescent signal output was measured by an off-line reader (BioTek, Synergy H1). Kinetics of the HRP-substrate reaction was measured at 425 nm every 47 s over a period of 10 min^34^. The resultant chemiluminescent signal is shown in Fig. 5a compared to the signal obtained by using the liquid chemiluminescent substrate without lyophilization. It is evident from the results that the single component substrate retains its functionality even after lyophilization and reconstitution which agrees with our previous work^34^. Similar experiments involving liquid chemiluminescent substrate and externally lyophilized and reconstituted DAb validated the functionality of the DAb post lyophilization. The HRP-conjugated DAb and the chemiluminescent substrate were lyophilized on the chip following the protocol, as shown in Fig. 6a. The COC MCFA lab chip with lyophilized reagents is shown in Fig. 6b with microscopic view of the lyophilization chamber.

The thermoplastic COC used for the fabrication of the MCFA lab chip has a relatively low surface energy with water contact angle of around 85°. The adequate hydrophilicity of the MCFA lab chips were obtained by treating the COC surface with a biocompatible hydrophilic coating P100 (Joninn, Denmark) which reduces the surface contact angle to around 12°. The fabricated COC chips were dip coated in the hydrophilic solution for 1 min and was immediately blown dry by Nitrogen gun generating a uniform coating with sufficient hydrophilicity. As reported in our previous work, the fundamental principle of protein adsorption on COC surface is through hydrophobic interactions with a low energy surface and there is very poor protein adsorption on hydrophilic COC surfaces^47–50^. Hence antibody incubation and blocking need to be performed on untreated and bare COC spiral reaction channels with no hydrophilic coatings. Accordingly, the MCFA lab chips were partially coated by keeping the reaction chamber part above the surface of the hydrophilic solution. Moreover, the spiral reaction chambers were masked by a water-impermeable tape and the inlets to the reaction chambers were blocked by photoresist during hydrophilic coating ensuring required hydrophobicity of the reaction area. A biocompatible hydrophobic coat A10 (Joninn, Denmark) was applied to create a patch in the sample loading chamber and was also applied to the circular pad of the vent connected to the substrate path. Finally, the MCFA lab chip was bonded by a thin film of COC (110 μm) to produce the final device.

In order to facilitate capillary flow in spiral reaction channels, the hydrophilic property of bovine serum albumin protein adsorption on COC surface was optimized. As reported earlier, BSA adsorbed polymer surfaces exhibit hydrophilic nature^51^ and hence capillarity of spiral reaction channels were controlled using blocking buffer which has 1% BSA in PBS. Untreated and flat COC chips (3 cm × 4 cm × 1.1 mm) were fully immersed in Phosphate buffer solution with BSA concentration of 10 mg/mL for different time periods ranging from 5 min to 45 min. At an interval of 5 min, BSA coated COC chips were taken out of the solution and vacuum-dried for 2 h at 100 mTorr. After 2 h of vacuum-drying, Artificial serum (AS) and DI water contact angle were measured on BSA coated COC chips using contact angle analyser. As evident from the results shown in Fig. 5b, AS contact angle decreased with increasing incubation time and reached a minimum for 30 min of immersion, followed by which there was a gradual increase in AS contact angle. The initial decrease in artificial serum (AS) contact angle with increase of BSA incubation was due to a monolayer coverage of BSA molecules with free polar groups, which facilitated wetting of polar liquids like AS and water. The increase in AS contact angle after 30 min of incubation can be attributed to the formation of double-layer of BSA which reduced the wettability of the surface by decreasing the surface energy available for adhesion^52^. Based on the results observed, optimized BSA concentration was fixed to be 10 mg/mL with an incubation time of 30 min which facilitated a high capillary flow in spiral reaction channels.

The LOC preparation steps thus can be summarized in the following steps. The injection molded COC chip was first solvent cleaned and then partially dip coated to achieve a low contact angle facilitating capillary flow. In total 6 μl of 0.8 μg/mL of HRP-conjugated detection antibody and 6 μl of the chemiluminescent substrate were added to the respective lyophilization chambers on the MCFA lab chip and were freeze dried for 18 h. The surface hydrophilicity and micropillar structures of the lyophilization chambers allow for easy spreading of the reagents and ensures uniform drying. Post-lyophilization, hydrophobic spots were created in desired places and the chip was solvent bonded producing a closed channel microfluidic device. Similar to the protocol followed in Optimiser^TM^ microfluidic microplate, optimized concentration of PfHRP2-CAb (100.0 µg/mL) and positive control CAb (40 μg/mL) was incubated in test and positive control spiral reaction channels, respectively, for 20 min and then drawn out. Then, all the three reaction chambers were blocked (1% BSA in PBS) for 30 min which helped to reduce non-specific binding and also increased the hydrophilicity of the reaction chambers. The chips were finally vacuum-dried for 2 h at 100 mTorr in Lindberg/Blue Vacuum Oven, VO1218A, (Thermo Fisher Scientific). Although hydrophilic coatings used were highly volatile, vacuum-drying ensured efficient removal of residual solutions and generated uniform coating thickness. Vacuum-dried COC chips were placed in ambient temperature conditions for 2 h after which artificial serum spiked with different concentrations of PfHRP2 antigen was added to the sample loading chamber.

On addition of the sample spiked with target antigen, the adequately hydrophilic polymer surface pulls the sample towards both the drying chambers. A part of the sample flowing into path-1 reconstitutes the HRP labeled detection antibody which binds to the target antigen forming the HRP–DAb-antigen complex and subsequently flows into the spiral reaction chambers. The other part of the sample flowing into path-2 reconstitutes the dried chemiluminescent substrate. The meandering channel in path-2 provides a high flow resistance and reduces the flow rate. As a result, the sample in path-1 reaches the reaction chambers first and consequently stops the flow in path-2 by trapping the air in between. The carefully designed stop valve prevents the backflow of the HRP–DAb-antigen complex into the substrate path and ensures that the enzyme-substrate reaction occurs only in the reaction chambers. The three spiral reaction chambers acted as test, positive and negative control to quantitatively determine the concentration of the target biomarker. The reaction chambers were connected in series at a pitch of 9 mm, same as 96 well plates to facilitate reading by a conventional benchtop reader. With the above-mentioned dimensions, the calculated surface-to-volume ratio was calculated to 180 cm^−1^ which is almost 18 times higher than that of a conventional 96 well plate. The high surface-to-volume ratio increases the rapidity and intensity of the reaction with much smaller reagent volume. The reconstituted HRP–Dab-antigen complex on reaching the test spiral chamber binds with the surface immobilized capture antibody and forms the desired capture antibody-antigen-detection antibody complex. The sample in path-1 continues to flow through the reaction chambers promoting washing of unbound reagents until the sample is drained from the left half of the sample loading chamber. The high capillary pressure of the microfluidic channels connecting the sample loading chamber to the Dab drying chamber prevents further draining of the sample and eventually stops the flow. The hydrophobic vent connected to path-2 is then opened which allows the reconstituted substrate to eventually reach the reaction chambers and the enzyme-substrate reaction in the test chamber produces the chemiluminescent light which can be directly correlated to the antigen concentration.

**Fig. 7:**
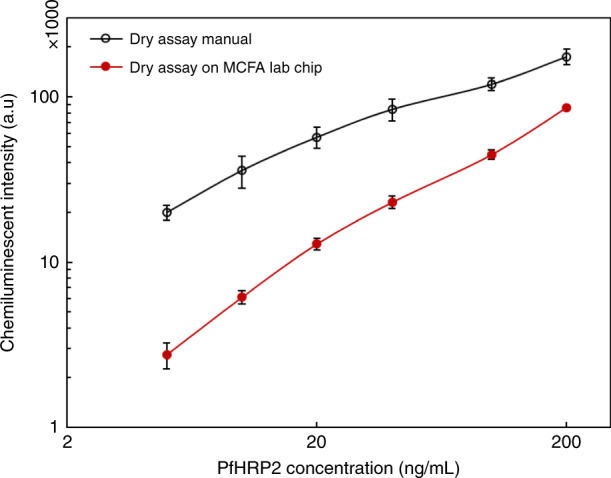
Variation of chemiluminescence intensity with variation in target antigen concentration. The black line signifies the assay results obtained with externally lyophilized and reconstituted reagents added to the reaction chambers manually. The red line on the other hand shows the assay results obtained from the MCFA chip with on-chip lyophilized reagents. The antigen concentration was varied from 5 ng/mL to 200 ng/mL

It was observed that after the vent was opened, it took around 1 min for the initiation of the chemiluminescence signal in the test chamber which remain saturated for the next 20–25 min. The output chemiluminescence measured from the test reaction chamber using a benchtop analyser (Synergy BioTek, H1) is shown in Fig. 7 along with the results obtained by manually performing the full assay with externally dried and reconstituted substrate and DAb in the reaction chambers. It was observed that there was a linear increase in chemiluminescence output signal with increase in PfHRP2 concentration from 5 ng/mL to 200 ng/mL with an inter-chip C.V of <10%. However, as evident from the results the sensitivity of the dry reagent immunoassay performed on the MCFA lab chip is less than when performed manually. That can be attributed to incomplete reconstitution and partial recovery of the dried reagents. Further implementation of delay in path-1 and path-2 will improve the reconstitution of the reagents and can improve the sensitivity of the functional chip.

### Assay results from MCFA lab chip on smartphone analyzer

**Fig. 8:**
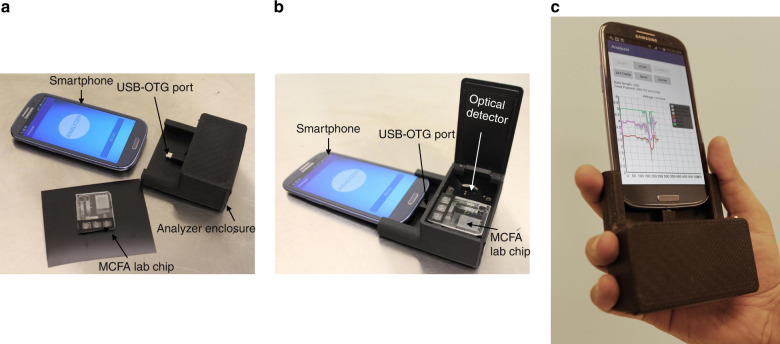
Step by step assembly of the smartphone based portable POCT analyzer. **a** Three major components such as MCFA lab chip, optical analyzer and smartphone, **b** Assembled parts with MCFA lab chip reaction chambers aligned with the photomultiplier and **c** functional hand-held POCT analyzer

The smartphone based portable analyzer system consists of an android smartphone (Samsung Galaxy SIII, Android Version 4.2.2), a low-level light detection circuit assembled together in a 3D printed black box, and the fabricated MCFA lab chip, as shown in Fig. 8. The optical detector detects the optical signals produced by chemiluminescence in the spiral reaction chambers of the MCFA lab chip, converts it into a digital form and transmits the data to the smartphone for processing and display. The smartphone powers the electronic circuit and communicates with it via an USB cable using the USB-OTG protocol. The smartphone acts as the host to the electronic interface device which acts as the USB peripheral. A customized android application was designed in the Android Studio IDE (Version 3.0.2) to facilitate user operation. The backend code is written using Java and the user interface is designed using XML.

**Fig. 9:**
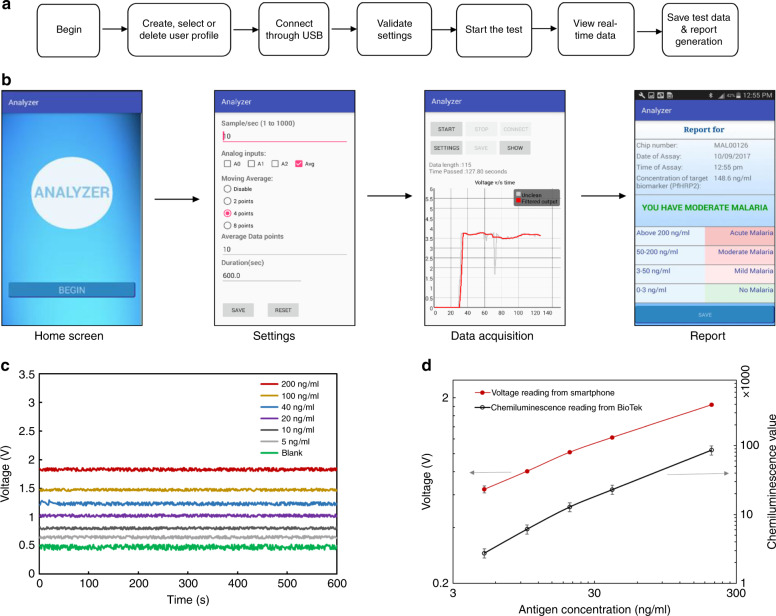
Android based smartphone application and assay results. **a** Flowchart depicting the architecture of the android application, **b** Application screenshots showing step by step operation of the smartphone analyzer, **c** The voltage vs time plot for different concentrations of malaria biomarker PfHRP2 as recorded by the smartphone and **d** the voltage variation with different concentrations of malaria biomarker PfHRP2 as recorded by the smartphone analyzer is plotted (red line) as compared to the chemiluminescent intensity recorded by a conventional reader for the same PfHRP2 concentrations

A flowchart depicting the interaction between the user and our system is shown in Fig. 9a. All operations are controlled by simple buttons guiding the user through a series of steps. For e.g., the first page introduces the user to the app, the next page provides step by step instructions on how to perform the test on the lab-on-a-chip, the next page allows the user to create, delete and select a user profile, the next allows the user to establish a connection with the interface device and then start the data collection process, and the last page displays the result of the test, as shown in Fig. 9b. When the peripheral device is connected to the smartphone, it follows an Attach Detection Protocol (ADP) where it detects a change in the VBUS capacitance at its USB port. On successful detection, it sends a signal to the applications listening to it. Once the connection is successfully established, the user can begin the data collection and analysis process by pressing the ‘Start’ button. On pressing the ‘Start’ button, the process of data reception and analysis begins. After string of data is received, it is parsed to extract the three data values, each corresponding to a voltage reading from the three analog ports of the Arduino which in turn corresponds to the optical signal generated by each of the reaction chambers on the MCFA lab chip. At a sampling frequency of 10 Hz, 10 samples of data are received per second from each port. The 10 samples are averaged over 1 s to give 1 data value per second for each port. The three averaged data values are then stored in three separate array lists: dataPoints0, dataPoints1 and dataPoints2 which are then used for plotting a voltage-time graph in real time. A separate ‘Settings’ page was created for adjusting parameters like sampling frequency, number of moving average filter points, test duration etc. There is also an option to save the results to a file in the smartphone which can later be accessed and presented to a medical practitioner or stored for future analysis. The sensitivity of the low-level light detection circuit was characterized by adding artificial serum spiked with PfHRP2 (5–200 ng/mL) into the sampling loading chamber like before.

After opening the hydrophobic vent connected to path-2, the chips were placed inside the black box with the reaction chambers aligned with the photomultipliers. Without the microfluidic chip, the voltage readings from all the 3 analog inputs were recorded to be around 350 mV and was considered as the base noise. The voltage reading corresponding to the positive control and negative control were ~3.1 V and 0.5 V, respectively and can be used for the quality check of the microfluidic chip and the system. The use of a 4-point moving average filter reduced the noise and generated a relatively smooth response profile, as shown in Fig. 9c. The use of a 2-point moving average is not sufficient to remove the noise in the signals whereas an 8-point moving average introduces substantial lag between the actual signal and the filtered signal. Considering the nature of chemiluminescence signal profile, data acquisition was made for a duration of 10 min which ensures saturation of the chemiluminescence signal.

Data points corresponding to the last 30 s were averaged to generate the voltage-concentration graph for the different concentrations of PfHRP2 and is shown in Fig. 9d. As shown in the results, the chemiluminescent intensity calculated from the portable analyzer and the conventional benchtop reader showed very similar trends. The limit of detection (LOD) was calculated from the results obtained from the smartphone analyzer and was calculated to be around 8 ng/mL which is comparable to other reported PfHRP2 immunosensors^18,53,54^. The PfHRP2 levels in clinical blood samples can range from as low as 10 ng/mL to as high as 1000 ng/mL^55^. The LOD for the MCFA lab chip was calculated as LOD = limit of blank (LOB) + 3*(SD for lowest concentration sample with a CL signal higher than blank), and the LOB was calculated as LOB = Mean blank response + 3*(SD_Blank), where SD is the standard deviation. Blank refers to the assay condition where addition of target antigen was replaced by blocking buffer addition ensuring an ideally zero concentration of biomarker. All analyte concentrations were analyzed in triplicate (3 chips). Considering the LOD, the MCFA lab chip with the analyzer can be considered as sensitive enough to detect an active malarial infection

## Conclusions

Microfluidic platforms for immunodiagnostics or molecular diagnostics have been a field of constant growth in the recent years. The realization of an ideal POCT system largely relies on the development of cheap and disposable microfluidic devices that can be easily integrated to low power electronics with a user- friendly interface. This paper reports the successful development of a hand-held POCT analyzer for detecting infectious diseases like malaria. Successful lyophilization and reconstitution of chemiluminescent substrate paved the way towards the design of a novel microfluidic chip. The reported device utilizes the higher sensitivity of chemiluminescence detection while eliminating the problems associated with liquid handling, storage, multiple pipetting and also shelf life. Although the shelf life characterization of the lyophilized reagents was not reported in this work and is in progress, lyophilized products in general have been reported to have a very long shelf life^56^. Further, the reported MCFA lab chip is one of the very first lab-on-a-chips to report chemiluminescence based sandwich ELISA controlled entirely by pure capillary flow. The designed microchannels and reservoirs of the MCFA lab chip produced a controlled and sequential flow of immunoassay reagents to a reaction area and utilized the high reaction kinetics of the spiral chambers to produce strong chemiluminescence reaction with a single sample loading step. Sandwich ELISA for the detection of malarial biomarker PfHRP2 was successfully performed on the MCFA lab chip with a clinically relevant LOD of 8 ng/mL which is sensitive enough to detect active malarial infection. The incorporation of positive and negative controls reduces the chance of false diagnosis and generates reliable and quantitative results. It is also evident from the results that the reconstitution and recovery of the dried reagents need to be further explored and the LOD can be significantly improved by the optimization. Introducing further delays in both the paths can help in a slower reconstitution leading to a better recovery and higher sensitivity. One of the limitations of the reported MCFA lab chip is that the device operation relies on the user to open the hydrophobic vent to initiate the chemiluminescence reaction. However, volume and the flow resistance in the substrate path can be optimized to control the time at which the reconstituted substrate reaches the reaction chamber making the entire operation from sample addition to detection automatic. Moreover, microchannel-based or membrane-based blood-plasma separator can be integrated into the MCFA lab chip for detection from whole blood. Future work will focus on developing a fully autonomous MCFA lab chip for biomarker detection from whole blood. The injection molded COC chips are inexpensive (e.g., chip fabrication cost can be estimated as around a few tens of cents considering the volume of production), disposable and suitable for biological assays. No requirement of any external flow control and chemiluminescence based detection (which does not require any excitation source) makes the lab chip easily integrable to a smartphone through an attachable/detachable optical analyzer. The use of the smartphone as a display device, data processor and power source paves the way towards a hand-held analyzer system for point-of-care-testing in resource limited environment. The reported MCFA lab chip with the smartphone analyzer can be easily customized and can provide a low cost, low power, portable platform for fast and sensitive detection of various biomarkers for disease diagnostics and prevention.

## Material and methods

### Fabrication of the MCFA lab chip

**Fig. 10:**
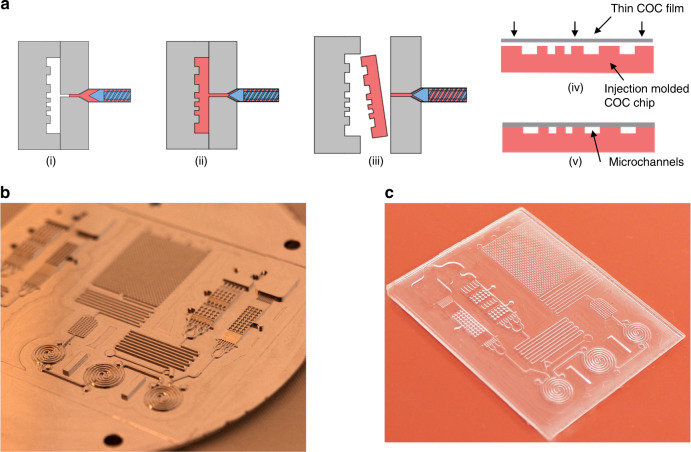
MCFA device fabrication. **a** Schematic of the COC LOC fabrication process: (i) clamping and plasticization, (ii) molding, (iii) LOC ejection, (iv) Solvent bonding using thin COC film, and (v) Solvent bonded MCFA lab chip. **b** Magnified image of the fabricated aluminum micro-mold on a 3-inch aluminum wafer and **c** Injection-molded MCFA lab chip on COC

The microfluidic chips reported in this work were fabricated from a high precision metallic micro-mold insert using injection molding^57^. The schematic of the microfabrication process is illustrated in Fig. 10a. A detailed 3-dimensional design of the mold was designed in the Mastercam^TM^ software and the machining toolpaths were created which can be directly fed to a computer numerical control (CNC) milling machine (Microlution 5100-S, Microlution Inc., IL) for micro-mold fabrication. Aluminum alloy (6061, Mcmaster Carr, USA) has been used as the master mold material since it is flexibly machinable and can run for several hundred replication cycles without deformation. This process facilitates microchannels of different depths to be incorporated in a single design, connected using ramp like structures to ensure a seamless flow through zones of variable capillary pressure. A 2° draft angle was used for the solid design to facilitate ejection during injection molding. The finished master-mold is shown in Fig. 10b. Cyclic Olefin Copolymer (COC) of grade 5013-S was obtained from Topas Advanced polymers (Kentucky, USA). COC was chosen because of its high affinity towards surface immobilization of antibodies, high resistance towards polar solvents and very high flow rates during injection molding^43^. COC chips shown in Fig. 10c were fabricated from the micro-mold by polymer injection molding using the injection molding machine (BOY 22 A Procan CT). The process cycle time was optimized to 35 s thus ensuring lower injection molding cost and increasing the throughput of the process.

The chips were laser cut, reagent loading ports were drilled and were solvent cleaned. The chips were then dip coated in a biocompatible hydrophilic coating solution (P100, Joninn, Denmark) to obtain a uniform contact angle of ~12° throughout the chip. While dip coating, the spiral reaction chambers were masked using water impermeable tapes to preserve the natural contact angle of ~85° in the reaction sites ensuring excellent protein adsorption. The enzyme-labeled detection antibody and the chemiluminescent substrate were lyophilized in the respective chambers on the open chip as explained earlier. The biocompatible hydrophilic coating prevents any non-specific binding in the lyophilization chambers and facilitates recovery of the dried reagents. The vent connected to the substrate path was rendered hydrophobic by adding a drop of hydrophobic coating (A10, Joninn, Denmark) in the circular pad. Finally, the chip was sealed using solvent bonding^58,59^. COC films of 110 μm thickness (8007S-04, Topas, USA) were used for the bonding purpose. The thin COC films were cleaned in 2-propanol and were dried with nitrogen. Three filter papers (Carl Schleicher & Schüll #. 589/3) were soaked with the solvent (75 mL of Cyclohexane mixed with 125 mL of Acetone) and in the “coating” step, the COC lid was placed on the filter papers for 2 min. Subsequently, the coated COC film was cleaned using acetone and dried with compressed air creating a tacky film which was pressed on to the patterned COC chips at a force of 900 pounds for 4 min in Wabash^TM^ pneumatic press producing the final lab chip.

### Assay reagents and optimization protocol

The sandwich ELISA for the detection of malaria biomarker PfHRP2, which followed the assay protocol described in Fig. 2a, was performed using anti-PfHRP2 capture antibody (MPFM-55A), HRP conjugated anti-PfHRP2 detection antibody (MPFG-55P) and purified PfHRP2 protein (AG55-0092-Z), all obtained from Immunology Consultants Laboratory (ICL), Inc., USA. Uniglow^TM^ one-component chemiluminescent (CL) substrate, Uniglow-0100 (Rockland antibodies and assay, USA) and QuantaRed^TM^ Enhanced Chemifluorescent (CF) HRP Substrate, 15159 (Thermo Fisher Scientific), were used to perform the CL-based and CF-based assays, respectively. AffiniPure Goat Anti-Mouse IgG,115-005-071, (Jackson ImmunoResearch laboratories, Inc. USA) was used as the positive control capture antibody. Elisa plate coating buffer, DY006 (R&D systems, USA) was used as the capture antibody diluent. Reagent diluent concentrate 2, DY995 (R&D systems, USA) was diluted with DI water to 1% BSA in PBS, filtered and used as the DAb diluent as well as the blocking buffer for the MCFA lab chip. Starting Block^TM^ 37538 (Thermo Fisher Scientific) was used as the blocking buffer in the Optimiser™ microfluidic microplate for assay optimization. Serasub™ (CST technologies, Inc., USA) is a liquid, protein free serum substitute for use in manufacturing clinical chemistry and immunological standards and controls and was used for spiking different concentrations of PfHRP2. A conventional 96-well plate reader, Synergy H1 (BioTek, USA), was used for reading the conventional 96-well plate, the Optimiser^TM^ microfluidic microplate, and the MCFA lab chip. A special holder was made to fit the benchtop reader dimensions in case of the LOC chip.

For optimizing antibody concentration to be used in microfluidic platform, Chemifluorescence based Malaria PfHRP2 detection was performed in Optimiser™ microfluidic microplate using all the assay reagents at a fixed concentration except for the antibody to be optimized. All ELISAs in Optimiser™ microfluidic microplate were performed based on the following protocol: 5.0 µl of CAb was incubated for 10 min, followed by blocking for 10 min using 5.0 µl StartingBlock^TM^ buffer. 5.0 µl sample and HRP-conjugated DAb was incubated consecutively for 10 min, followed by a plate wash using 30.0 µl of phosphate buffer solution (PBS, pH 7.2–7.4). Then, 10 µl of QuantaRed^TM^ Enhanced Chemifluorescent (CF) substrate was added and the plate was read after 15 min. 800 ng/mL of Malaria PfHRP2 antigen concentration (assay buffer) was used for all optimization assays.

### Design and implementation of optical detector

Silicon photomultiplier sensors (MicroFC-30035-SMT-C1) were used for the chemiluminescent light detection and were obtained from ON Semiconductors (Phoenix, AZ). The transimpedance amplifiers (OPA320) were from Digi-Key (Thief River Falls, MN). Teensy 3.2 microcontrollers were from PJRC.com, LLC. (Sherwood, OR). The analog to digital convertors (ADS111), unregulated DC-DC converters (DCV010512D) and 3-terminal adjustable regulator (LM337L) were obtained from Texas Instruments Inc. (Dallas, TX). Other Passive electrical components (capacitors, resistors, contact pins, wires) were from Digi-Key (Thief River Falls, MN). The interface box was designed in SolidWorks software and 3D printed in the Digital Fabrication Lab at University of Cincinnati. The box is made of ABS plastic and printed on the Dimension SST 1200es^TM^ printer by Stratasys. The electronic system was developed to enable automated detection of target biomarker from the chemiluminescence assay performed in the MCFA lab chip and enable user interaction with the system through a smartphone. Main functions include measurement of low-level optical signal generated out of assay, converting optical signal from analog to digital form and interacting with USER commands via smartphone.

**Fig. 11:**
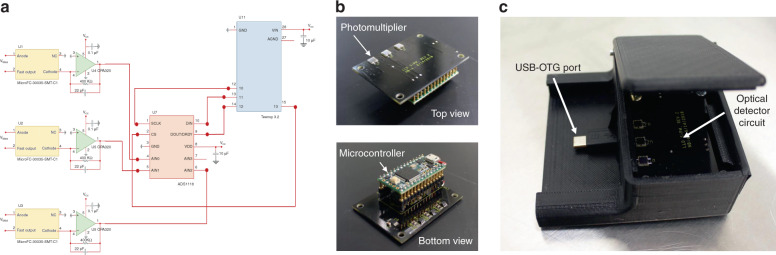
Low-level light detection circuit. **a** Schematic of the optical detection circuit, **b** Implemented optical detector on designed PCB illustrating the photomultiplier on the top side and the microcontroller on the bottom side, and **c** Optical detector circuit with USB-OTG cable assembled inside a 3D printed enclosure for attachment to the smartphone

This system comprises of highly sensitive photo multiplier, 16-bit sigma-delta analog to digital converter, 8-bit microcontroller and a smart phone device. Circuit comprising above mentioned components was designed using OrCAD schematic editor and a custom printed circuit board (PCB) was designed using OrCAD PCB designer. PCB was fabricated by PCB Prime (Aurora, CO) and assembled internally. The schematic of the low-level light detection circuit is shown in Fig. 11a. Circuitry design starts with a highly sensitive photo multiplier chip (MicroFC-30035-SMT-C1) with an internal gain of ~1E + 9 followed by a transimpedance amplifier OPA320 with a gain of 400 kV/A. Operation amplifier output is a single-ended voltage signal with a swing of 0–5 V. Following the amplifier, an analog to digital converter chip (ADS1118) converts the signal from analog voltage (0–5 V) to digital signal (16-bit). Measured data in digital format is then transferred from ADS1118 to a microcontroller (TEENSY 3.2) via SPI serial interface. Microcontroller acting as a central processing unit, receives/transfers the measurement data from the detection circuit (ADS1118) to the smartphone via serial interface. A printed circuit board with an outer dimension of 38 × 50 mm was designed using OrCAD PCB Designer. For high signal-to-noise (SNR) ratio, highly sensitive optical signal detection circuit was placed on the top side, whereas rest of the circuit components were placed on bottom side of the PCB. The developed optical detection circuit is shown in Fig. 11b and the overall assembly of the detector in the enclosure is shown in Fig. 11c. All passive components (resistors and capacitors) were chosen to be of 0603 package.

Minimum trace width used in the design is 127 µm, which can carry electrical current up to 350 mA. Which is far enough for the signals used in the interconnect routing. Circuit is powered through 5 V supply, trace width used to carry power across the board is 508 µm, which can carry ~1.12 A. Entire circuit is USB bus powered at 5 V. Maximum rated power consumption is 0.5 W which is 100 mA at 5 V supply. Except the TEENSY 3.2 microcontroller, all other components are surface mount package. The analyzer system in this project, has been developed using a Samsung Galaxy SIII which has a 2100 mAh battery. The system consumes up to 6% of the smartphone’s battery in 10 min when interfaced with the USB OTG-based device, thus over 16 diagnostics of malaria samples are continuously possible without re-charging the smartphone.
